# Supplementary material for: PANGEA: a new gene set enrichment tool for Drosophila and common research organisms
Source: Nucleic Acids Res. 2023 May 1;51(W1):W419–26. doi: 10.1093/nar/gkad331 (PMC10320058; doi:10.1093/nar/gkad331)

Supplementary Figure 1: Exporting a list of Drosophila genes from FlyBase to PANGEA. A 'Hitlist' in FlyBase is a list of class entities (e.g. genes, alleles, transcripts). The top section shows the ways in which a HitList can be generated – via query tools such as 'Vocabularies' and 'QuickSearch', via user input using the ID validator tool or via the 'Export to HitList' button found on many FlyBase pages containing lists such as Gene Group and Pathway pages. From the HitList (bottom section), entities such as transcripts and alleles can be converted to a gene list via the 'Convert' dropdown menu. A list of genes can be sent directly to PANGEA via the 'PANGEA Enrichment Tool (DRSC)' option on the dropdown 'Export' menu (inset).

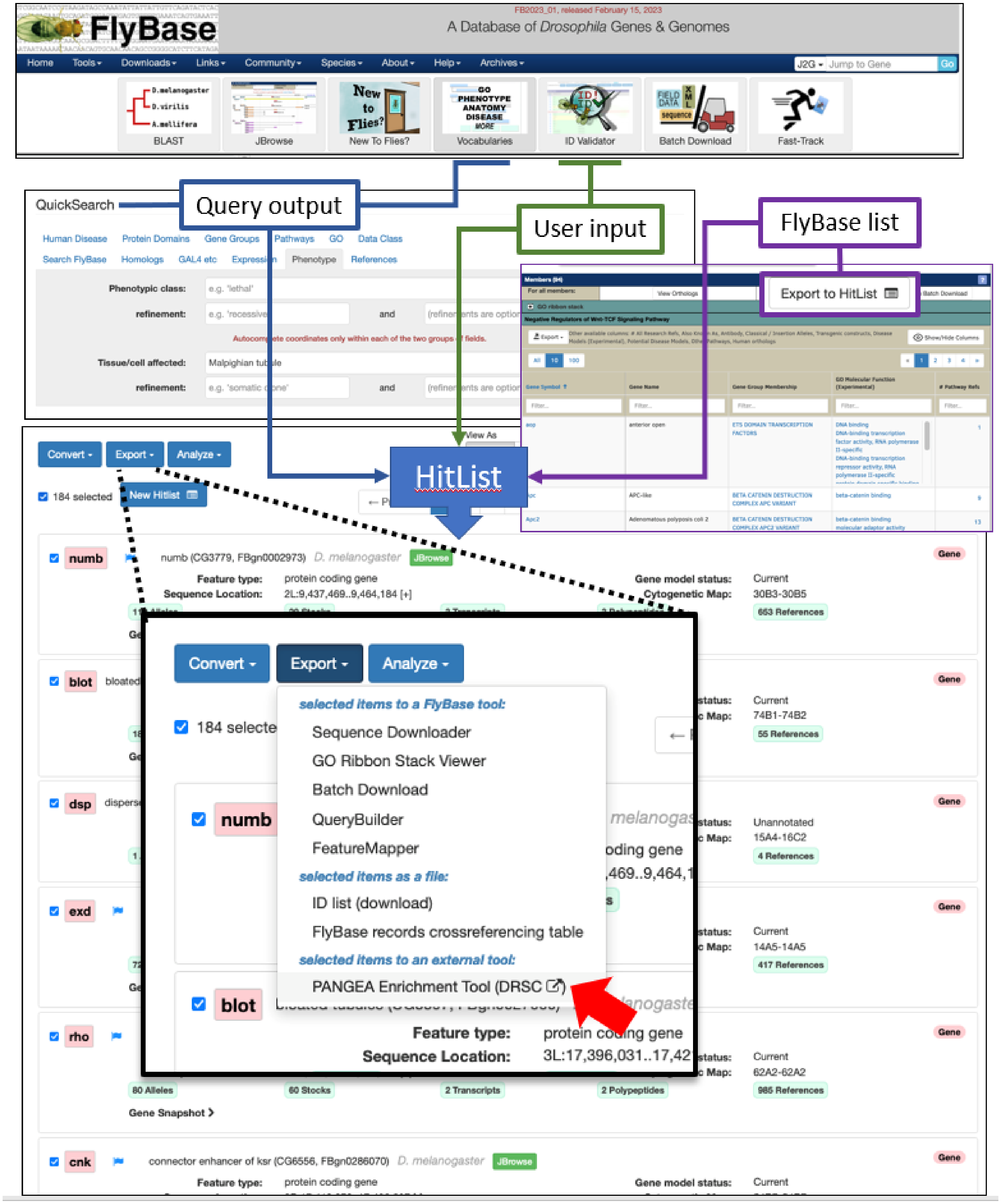

Supplement: gkad331_Supplemental_Files [file gkad331_supplemental_files.zip › supplementary_figure1.pdf]
