# Supplementary material for: PANGEA: a new gene set enrichment tool for Drosophila and common research organisms
Source: Nucleic Acids Res. 2023 May 1;51(W1):W419–26. doi: 10.1093/nar/gkad331 (PMC10320058; doi:10.1093/nar/gkad331)

Supplementary Figure 2: The proteomic dataset of high-confident preys of four bait proteins (Fl(2)d, METTL14, METTL3, Nito) was obtained from the supplementary table 2 of the published study by Tang et al (PMID: 33649236, https://www.ncbi.nlm.nih.gov/pmc/articles/PMC7958400/). PANGEA "Search Multiple" function was used and Phenotype annotation was selected. The comparison of the enrichment of *Drosophila* phenotype annotation from the interacting proteins of four different baits was illustrated using a heatmap.

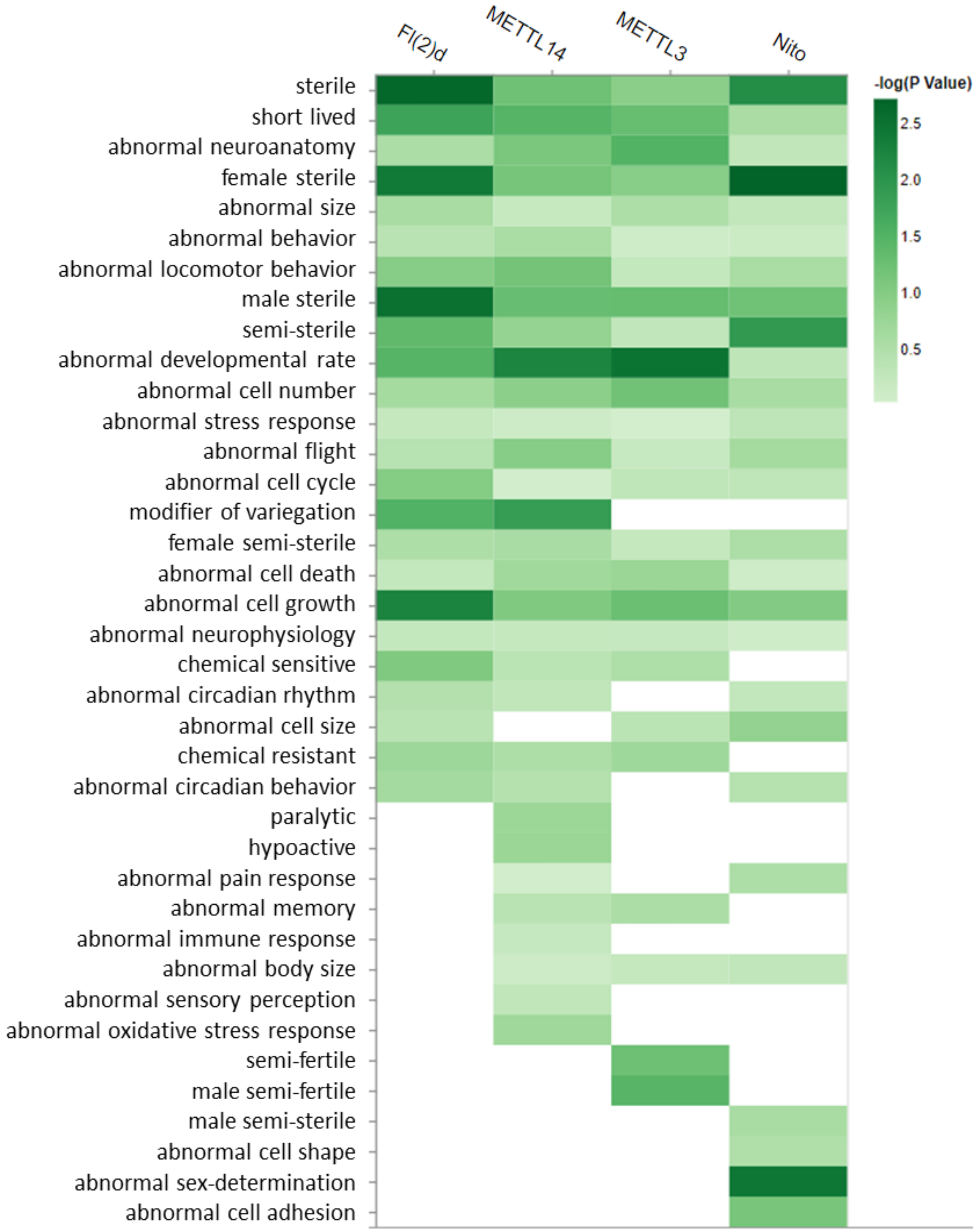

Supplement: gkad331_Supplemental_Files [file gkad331_supplemental_files.zip › supplementary_figure2.pdf]
